# Tissue-specific requirement of sodium channel and clathrin linker 1 (Sclt1) for ciliogenesis during limb development

**DOI:** 10.3389/fcell.2022.1058895

**Published:** 2022-11-03

**Authors:** Hankyu Lee, Kyeong-Hye Moon, Jieun Song, Suyeon Je, Jinwoong Bok, Hyuk Wan Ko

**Affiliations:** ^1^ Department of Biochemistry, College of Life Science and Biotechnology, Yonsei University, Seoul, South Korea; ^2^ College of Pharmacy, Dongguk University-Seoul, Goyang, South Korea; ^3^ Department of Anatomy, Yonsei University College of Medicine, Seoul, South Korea; ^4^ Brain Korea 21 (BK21) Project for Medical Science, Yonsei University College of Medicine, Seoul, South Korea; ^5^ Brain Korea 21 (BK21) FOUR Program, Yonsei Education and Research Center for Biosystems, Yonsei University, Seoul, South Korea; ^6^ Department of Otorhinolaryngology, Yonsei University College of Medicine, Seoul, South Korea

**Keywords:** primary cilia, distal appendage, limb development, SCLT1, ciliogenesis, ciliopathy

## Abstract

Primary cilia have essential roles as signaling centers during development and adult homeostasis. Disruption of ciliary structure or function causes congenital human disorders called ciliopathies. Centriolar distal appendage (DAP) proteins are important for anchoring cilia to the membrane. However, the exact functions of DAP during *in vivo* ciliogenesis and animal development remain poorly understood. Here, we showed that the DAP component sodium channel and clathrin linker 1 (Sclt1) mutant mice had abnormal craniofacial and limb development with postnatal lethality. In mutant embryos, most of the affected tissues had defects in DAP recruitment to the basal body and docking to the membrane that resulted in reduced ciliogenesis and disrupted hedgehog (Hh) signaling in limb bud mesenchymal cells. However, limb digit formation and ciliogenesis in *Sclt1* mutant mice were differentially affected between the fore- and hindlimb buds. The forelimbs developed normally in *Sclt1* mutants, but the hindlimbs had preaxial polydactyly. Heterozygous loss of *Cep83*, another core DAP component, in *Sclt1* mutant mice, caused forelimb and hindlimb polydactyly. These findings revealed the tissue-specific differential requirement of DAPs. Taken together, these results indicated that during limb development the ciliary base components, DAPs, play an essential role in ciliogenesis and Hh signaling *in vivo* in a position-dependent manner.

## Introduction

Most mammalian cells possess a primary cilium, which is a hair-like structure that emanates from the plasma membrane surface (Sorokin, 1962; Eggenschwiler and Anderson, 2007; Ko, 2012). The primary cilium contains a ciliary axoneme, 9 + 0 microtubule doublets, encapsulated within a specialized membrane. It also contains an unknown barrier structure in the basal body which is located in the proximal region of the cilium (Reiter et al., 2012). These components ensure that the primary cilium is discretely located from the other cellular compartments, and it functions as a cellular organelle. Although primary cilia are preserved as distinct membrane-associated structures in non-cycling cells, in dividing cells they cycle between the disassembly and re-assembly, depending on the stage of the cell cycle (Tucker et al., 1979). Disassembly resorbs the axoneme and ciliary membrane, leaving the basal body to form a centrosome that functions as a microtubule organizing center that regulates cell-cycle progress in proliferating cells. Re-assembly of primary cilia, or ciliogenesis ensues as cells enter the G0/G1 phase of the cell cycle. During ciliogenesis, the centriole progresses to a basal body with multiple steps of maturation, such as migration and docking into the membrane, followed by the growth of the axoneme from the basal body (Wang and Dynlacht, 2018). The molecular basis of centriolar docking to the plasma membrane was recently revealed (Tanos et al., 2013; Yang et al., 2018). The centriolar distal appendage (DAP) structure in the primary cilium forms a triangular filamentous base attached to the triplet microtubules of the centrioles and is important for basal body docking. DAPs are currently identified as composed of ANKRD26, CEP83, CEP89, CEP164, FBF1, LRRC45, PIDD1, and sodium channel and clathrin linker 1 (SCLT1); these components are recruited to the centriole in a hierarchical manner to assemble the DAPs (Tanos et al., 2013; Kurtulmus et al., 2018; Evans et al., 2021; Blanco-Ameijeiras et al., 2022). Removal of DAP genes results in reduced ciliogenesis, due to disruption of basal body docking.

Primary cilia have essential roles as signaling centers during animal development and adult homeostasis. They have been found as a signal processing center for hedgehog (Hh) signaling during mouse neural tube development (Eggenschwiler and Anderson, 2007; Goetz and Anderson, 2010). Activation of the pathway *via* binding Hh ligand to the receptor, Patched (Ptch), displaces it from the ciliary compartment, and leads to accumulation of the seven-transmembrane receptor, Smoothened (Smo), in the ciliary membrane to derepress Hh signal transduction. Gli transcription factors, which are downstream effectors of Smo in the Hh pathway translocate to the tip of the ciliary axoneme to transform into an active form with an unknown mechanism. They then enter the nucleus to propagate Hh signaling.

Results from human genetic studies indicate the importance of proper formation of ciliary structures and functions in human genetic disorders (Reiter and Leroux, 2017). Disruption of ciliary genes related to ciliogenesis results in malformation of multiple organs in humans, known as ciliopathies. Clinical symptoms in these syndromic disorders include defects particularly in the brain, eye, kidney, and bone development (Badano et al., 2006). Skeletal abnormalities and associated symptoms are frequently found in ciliopathies that include skeletal dysplasia and limb digit patterning defects. Animal model systems for affected genes recapitulate the corresponding clinical features of ciliopathies in humans. Studies from these model systems suggest that malformations of primary cilia cause disruption of Hh signaling during development, leading to skeletal ciliopathies. However, the exact underlying mechanisms for skeletal ciliopathies are poorly understood.

The basic structure and genes of cilia are well conserved throughout the species. The DAPs of the centriole are a crucial substructure required for anchoring the centriole (i.e., the cilium organizing center) to the membrane. The centriole and associated DAPs are a good example of evolutionary conservation. However, results of mouse and human studies of DAP genes such as Cep83, Cep164, and FBF1 indicate that gene-dependent variation in phenotype that includes defects in ciliogenesis, can occur (Failler et al., 2014; Siller et al., 2017; Zhang et al., 2021). Here, to elucidate the role of SCLT1 in ciliogenesis and reveal the molecular basis of associated human diseases using a mouse model system. We analyzed ciliogenesis and developmental abnormalities in Sclt1-disrupted mouse embryos. We found that mutant embryos display common features of ciliopathies in selected tissues, such as the palate and limbs, which were affected by cleft palate and polydactyly, respectively. These results are consistent with the phenotypic abnormalities found in transgenic allele of Sclt1 (Li et al., 2017). Most affected tissues had loss of ciliogenesis that led to a diminished response to Hh signaling. Limb digit number patterning defects in *Sclt1* mutant embryos were restricted to the hindlimbs; forelimb development was normal. We also found that a double mutant embryo combination of SCLT1 null and Cep83 heterozygote had both forelimb and hindlimb polydactyly. These findings suggested that evolutionarily conserved ciliary base components have roles in ciliogenesis and Hh signaling *in vivo* but that tissue-specific diverse ciliogenesis programs might exist.

## Materials and methods

### Mouse strains

*Sclt1* ^*tm1a(EUCOMM)Hmgu*^ (*Sctl1* ^*tm1a*^) and *Cep83* ^*tm1(KOMP)/Tcp*^ (*Cep83* ^*KO*^) mice were generated by obtaining embryonic stem (ES) cells targeted in the Sclt1 and Cep83 locus from the trans-NIH Knock-Out Mouse Project (KOMP). To produce targeted mice, ES cells were further processed for blastocyst injection and chimera production (Macrogen, Seoul, Korea). Chimeric mice were bred with C57/BL6N wildtype mice to confirm the germline transmission. Genotypes were performed using PCR with appropriate primers flanking the targeted region of Sclt1 and Cep83. *Sclt1* ^*KO*^ is a null allele generated from E2a Cre-mediated recombination of loxP sites flanking the exon 5 of the gene, and this allele was confirmed by sequencing. All mice were housed and bred in C57/BL6N background in a specific-pathogen-free animal facility with constant temperature and humidity and *ad libitum* access to food and water. All mouse experiments, housing, and breeding protocols were approved by the Institutional Animal Care and Use Committee of Dongguk and Yonsei University, Korea.

### Cell culture

For primary mesenchymal cell culture, limb buds were dissected from E11.5 mouse embryos and then digested with dispase II to dissociate cells. After seeding the cells in 24-well plates, differentiating media (DMEM/F12 media containing 10% FBS, 50 μg/ml ascorbic acid, 10 mM β-glycerol phosphate, and 1% L-glutamine) was added. Cells were grown for 1 day and replaced with DMEM media for 30 h to induce ciliogenesis. Further experiments were performed after cilia induction.

### Western blotting

Whole embryo lysates for GLI3 or SCLT1 immunoblotting were obtained by clarifying E10.5 embryos in modified RIPA buffer (150 mM NaCl, 50 mM Tris-HCl [pH 7.4], 1 mM EDTA, 1% NP40, 0.25% Na-deoxycholate) with a protease inhibitor cocktail (Roche) for 15 min at 17,000 × g at 4°C. Protein concentration was determined with the BCA Protein Assay (Thermo Fisher Scientific). Equal amounts of protein (10 μg) were loaded for electrophoresis. After electrophoresis, proteins were transferred to PVDF membranes (Merck Millipore) using a Trans-Blot SD semi-dry transfer cell (Bio-Rad). Membranes were washed with Tris-buffered saline solution containing 0.2% Tween 20 (TBST) and blocked for 1 h in TBST containing 5% skim milk. Membranes were then incubated overnight at 4°C with a mouse monoclonal anti-GLI3 antibody (kindly provided by Dr. Susie Scales, Genentech, CA) for GLI3 processing or in-house generated rabbit polyclonal anti-SCLT1 antibody for SCLT1 detection. β-actin (Cell Signaling Technology) or α-tubulin antibody (Sigma Millipore) were used for internal control. Signals were detected with a chemiluminescent reagent (Merck Millipore) following the manufacturer’s instructions.

### Quantification of relative gene expression

Total RNA was isolated from E11.5 embryo or cultured primary limb mesenchymal cells using the TRI reagent (Invitrogen), and 1 μg of total RNA was reverse transcribed using an oligo-dT primer and a First-Strand cDNA Synthesis kit (Takara). Real-time PCR was performed using the Rotor Gene Q instrument (Qiagen) with SYBR (Takara). Beta-actin was used as an internal control. Results were analyzed with the Rotor Gene Q series software to calculate the relative mRNA levels using the 2^−*ΔΔCT*^ method.

### Immunofluorescent staining

To analyze the ciliogenesis in limbs, E11.5 embryo limbs were dissected in ice-cold 1 × PBS, fixed in 4% PFA, and prepared for cryosectioned samples using standard protocols. Cryosections were stained with the indicated primary antibodies and fluorophore-conjugated secondary antibodies, followed by DAPI counterstaining. Primary antibodies were rabbit anti-ARL13B, and anti-γ-tubulin (γ-Tub, Sigma Millipore). The secondary antibodies used were species-specific antibodies conjugated with AlexaFluor 488 or 594 (Jackson Immunoresearch). Ciliary frequency was measured using a LSM 880 confocal microscope (Carl Zeiss) and ZEN Software. Statistical analysis was performed using Student’s *t*-tests (two-tailed, equal variance).

For centriolar localization of DAP proteins in limb buds derived mesenchymal cells, anti-CEP83 (Sigma Millipore), anti-CEP89 (Sigma Millipore), anti-CEP164 (Sigma Millipore), and anti-FBF1 (Proteintech) antibodies were used. Cells were fixed with ice-cold methanol for 5 min and followed by standard protocols for immunofluorescent co-staining with appropriate primary antibodies and anti-γ-tubulin antibody (γ-Tub, Sigma Millipore).

### Limb skeletal staining

For limb skeleton, alcian blue and alizarin red staining was performed using standard procedures. Briefly, embryos at stage E18.5 were eviscerated and followed by hot tap water (65–70°C) treatment. Embryos were fixed in 95% ethanol overnight and then transferred to acetone overnight to remove fat. Embryos were then transferred into 0.03% alcian blue (Sigma Millipore, MO, United States) solution for cartilage staining. After a few washes with 70% ethanol for 6–8 h, samples were placed in 1% KOH until they became transparent. Next, for bone staining, embryos were transferred into 0.05% alizarin red (Sigma Millipore, MO, United States) overnight. Embryos were cleared by placing in 1% KOH/20% glycerol for 2 days and stored in a 1:1 mixture of glycerol and ethanol until image processing.

### Transmission electron microscopy

For transmission electron microscopy, limb buds of E11.5 embryos were isolated and fixed with 2.5% glutaraldehyde and 2% paraformaldehyde in 0.1 M sodium cacodylate buffer (pH 7.4) at 4°C overnight. The specimens were washed three times for 30 min in 0.1 M sodium cacodylate buffer (pH 7.4). Then the specimens were post-fixed with 1% osmium tetroxide at 4°C for 2 h, dehydrated using graded ethanol, and infiltrated with propylene oxide. Specimens were embedded using Poly/Bed 812 kit (Polysciences). After fresh resin embedding and polymerization in an electron microscope oven (DOSAKA, Japan) at 60°C for 24 h, the specimens were initially sectioned in 350 nm thickness using LEICA EM UC-7 Ultra-microtome (Leica Microsystems) and stained with toluidine blue for light microscope. The specimens were then sectioned in 80 ㎚ thickness and double stained with 7% (20 mins) uranyl acetate and lead citrate for contrast staining. All the thin sections were observed by transmission electron microscope (JEM-1011, 80Kv JEOL, Japan) at the acceleration voltage of 80 kV.

## Results

### Genetic disruption of the sodium channel and clathrin linker 1 (Sclt1) gene causes multiple developmental defects including hindlimb-specific preaxial polydactyly

**FIGURE 1:**
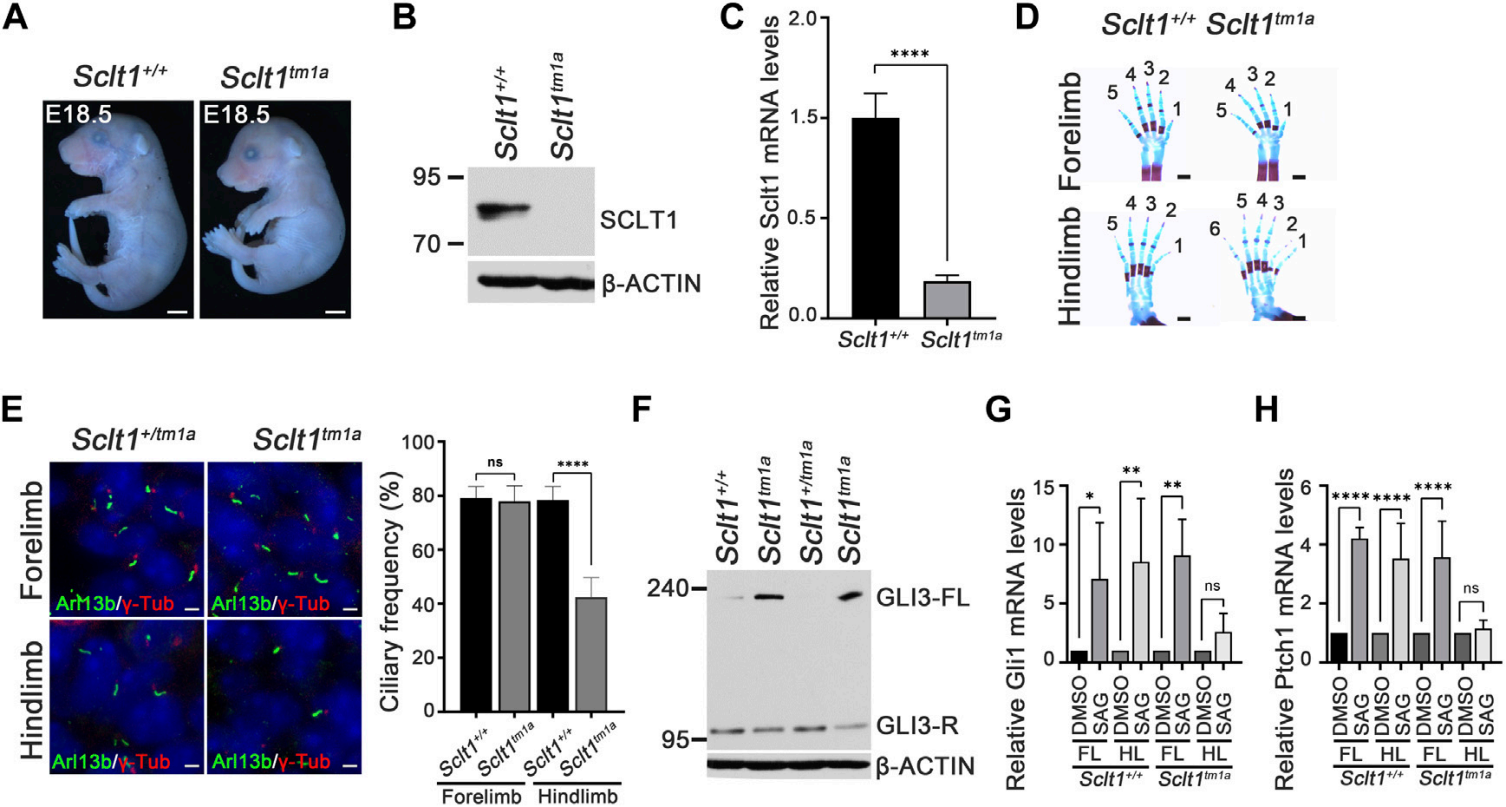
*Sclt1* mutant mice display hindlimb-specific preaxial polydactyly caused by disruption of ciliogenesis and Hh signaling **(A)** Gross morphological analysis of *Sclt1* ^*tm1a*^ and wildtype control littermates at stage E18.5. Scale bars = 1 mm **(B)** Western blot analysis to detect levels of SCLT1 from E10.5 whole embryo extracts. Signal specific to anti-SCLT1 antibody is undetectable in *Sclt1* mutant embryos, compared with wildtype control **(C)** Quantitative RT-PCR (qRT-PCR) analyses with RNAs from whole embryos at E10.5 confirmed that expression levels of properly spliced full-length transcripts were lower than those of wildtype transcripts **(D)** Alcian blue/alizarin red staining of limbs in E18.5 embryos. Hindlimbs of *Sclt1* mutant had abnormal digit patterning such as preaxial polydactyly **(E)** Left panel, representative immunofluorescence image of co-staining with ciliary marker, Arl13b (Arl13b, green), and basal body marker, gamma-tubulin (γ-Tub, red). Right panel, quantitative analysis of ciliogenesis from immunofluorescent stained *Sclt1* mutant and control limb buds. Error bars represent SEM and asterisk denotes statistical significance, based on Student′s *t* test results (***p <0.001) **(F)** GLI3 processing was analyzed using western blotting with anti-GLI3 antibody. Whole embryo extracts from E10.5 wildtype and *Sclt1* mutant embryos were obtained and then examined to detect the full-length (GLI3-FL) and processed (GLI3-R) forms of GLI3 protein with anti-GLI3 antibody. Two independent embryos for each genotype were used and β-actin levels were used as a control **(G)** To analyze the response to Hh activation in cultured primary mesenchymal cells of forelimb and hindlimb buds, 100 nM Smoothened agonist (SAG) were treated, and then qRT-PCR analysis was performed to measure mRNA levels of Hh target genes, Gli1 and Ptch1. Error bars represent SEM and asterisk denotes statistical significance, based on Student′s *t* test results (*p <0.05; **p <0.01; ****p <0.0001).

To analyze the effects of Sclt1 gene loss on mouse development, we generated a targeted mutation allele of the Sclt1 gene, *Sclt1* ^*tm1a(EUCOMM)Hmgu*^ (*Sctl1* ^*tm1a*^) by obtaining mouse embryonic stem cells containing the exon 5 region of the Sclt1 gene locus trapped with a β-galactosidase reporter tagged insertional cassette from the International Mouse Phenotyping Consortium (IMPC). We found that the gross morphology of *Sclt1* mutant embryos showed normal appearance in the prenatal stage but showed lethality with complete penetrance at postnatal day 0 (P0) (Figure 1A). We used protein-specific antibody to evaluate whether the targeted mutation affected SCLT1 protein levels. Western blot analysis for SCLT1 revealed an approximately 80 kDa band in wildtype (*Sclt1* ^*+/+*^) embryo extracts but not in homozygote mutant (*Sclt1* ^*tm1a*^) extracts (Figure 1B). This result indicates that the trapped reporter cassette affected gene expression in the mutant mouse embryo. We confirmed the disrupted gene expression using quantitative RT-PCR (qRT-PCR) analysis (Figure 1C). There was a more than 90% reduction in Sclt1 mRNA levels in homozygote *Sclt1* ^*tm1a*^ embryos compared with the wildtype. We also checked the expression pattern of Sclt1 by staining gene trapped lacZ activity in *Sclt1* ^*+/tm1a*^ and *Sclt1* ^*tm1a*^ embryos. LacZ was highly expressed in the eye, neural tube, and limb buds at stage E10.5 (Supplementary Figure S1).

Recently, human mutation of SCLT1 has been known to cause oral-facial-digital syndrome type IX in which defects are manifested as in a midline cleft of palate, microcephaly, and coloboma (Adly et al., 2014). To determine whether the same changes occurred in the mouse model system, we examined the phenotypic abnormalities in *Sclt1* ^*tm1a*^ embryos. Although the mutant embryos had normal development of the eye and other organs, they had defects in intestine, kidney, limb, and palate development (Figure 1D and Supplementary Figure S2). The *Sclt1* ^*tm1a*^ embryo showed the formation of a shortened intestine, cystic kidney, and cleft palate which are consistent with previous study from intragenic insertional allele of *Sclt1* (Li et al., 2017). These changes were similary observed in SCLT1 truncating mutation of human (Adly et al., 2014). We also found abnormal limb digit patterning with preaxial polydactyly in mutant embryos (Figure 1D). The forelimb developed normally, but the hindlimb was specifically affected by the *Sclt1* gene disruption. Due to an incomplete splicing event in knockout (KO) first strategy of tm1a allele, we used western blot to check for leaky expression of Sclt1 in the forelimb and hindlimb of *Sclt1* ^*tm1a*^ embryos (Supplementary Figure S3). Using SCLT1 specific antibody, we found that the signal was not detectable in mutant forelimb or hindlimb bud extracts. These results indicate that Sclt1 expression in the forelimbs and hindlimbs decreased in a comparable manner. These results suggest that Sclt1 differentially regulates limb digit patterning between the forelimbs and hindlimbs.

### Sclt1 regulates hindlimb-specific ciliogenesis during limb development

Sclt1 is a part of the centriolar DAP. It is involved in anchoring the mature centriole/basal body to the membrane. We examined the formation of primary cilia using immunofluorescent staining with Arl13b and gamma-tubulin (γ-Tub) antibodies (i.e., ciliary membrane, and basal body markers, respectively) in developing limb mesenchymal cells of E11.5 stage forelimbs (Figure 1E). In the control limb, Arl13b-positive cilia normally extended from the basal body stained with γ-tub antibody. Although primary cilia were normally present in the mutant forelimbs, the hindlimbs of *Sclt1* ^*tm1a*^ embryo had about a 50% reduced ciliogenesis compared with the control. These results indicate that Sclt1 in the developing hindlimb regulates the ciliogenesis, and loss of Sclt1 results in abnormal hindlimb specification in *Sclt1* mutant embryos.

Hedgehog (Hh) signaling is a primary signaling pathway affected by dysfunction of primary cilia (Eggenschwiler and Anderson, 2007; Goetz and Anderson, 2010). We examined changes in Hh signaling activity in developing mouse embryos. Activation of Hh signaling *via* Hh ligand binding to PTCH1 induces the GLI proteins processing. Full-length GLI3 is normally cleavaged without pathway activation to generate a repressor form of GLI3. Hh binding to PTCH1 blocks the processing of GLI3 and increases the levels of the full-length form of GLI3. We examined the GLI3 processing from E10.5 whole embryo extracts as described previously (Lee and Ko, 2020). Full-length GLI3 proteins accumulated in the mutant embryos with an decrease of repressor form, indicating that GLI3 processing is abnormally regulated by loss of Sclt1 (Figure 1F). In limb, the occurrence of Hh pathway activity in a concentration gradient manner is critical for specification of digit identity. We also examined the Hh response using qPCR analysis of primary mesenchymal cells obtained from forelimb or hindlimb buds. Activation of the Hh pathway by treating Smoothened agonist (SAG) in limb buds derived mesenchymal cells increased GLI transcription activity by upregulating Gli1 and Ptch1 mRNA expression (Figure 1G). In contrast, the response to SAG in mesenchymal cells from mutant hindlimbs was significantly impaired. These results suggest that Hh signaling in the *Sclt1* mutant embryo is ablated by reduced ciliogenesis.

### Sclt1 is involved in the hierarchical DAP assembly process during ciliogenesis in the hindlimb

**FIGURE 2:**
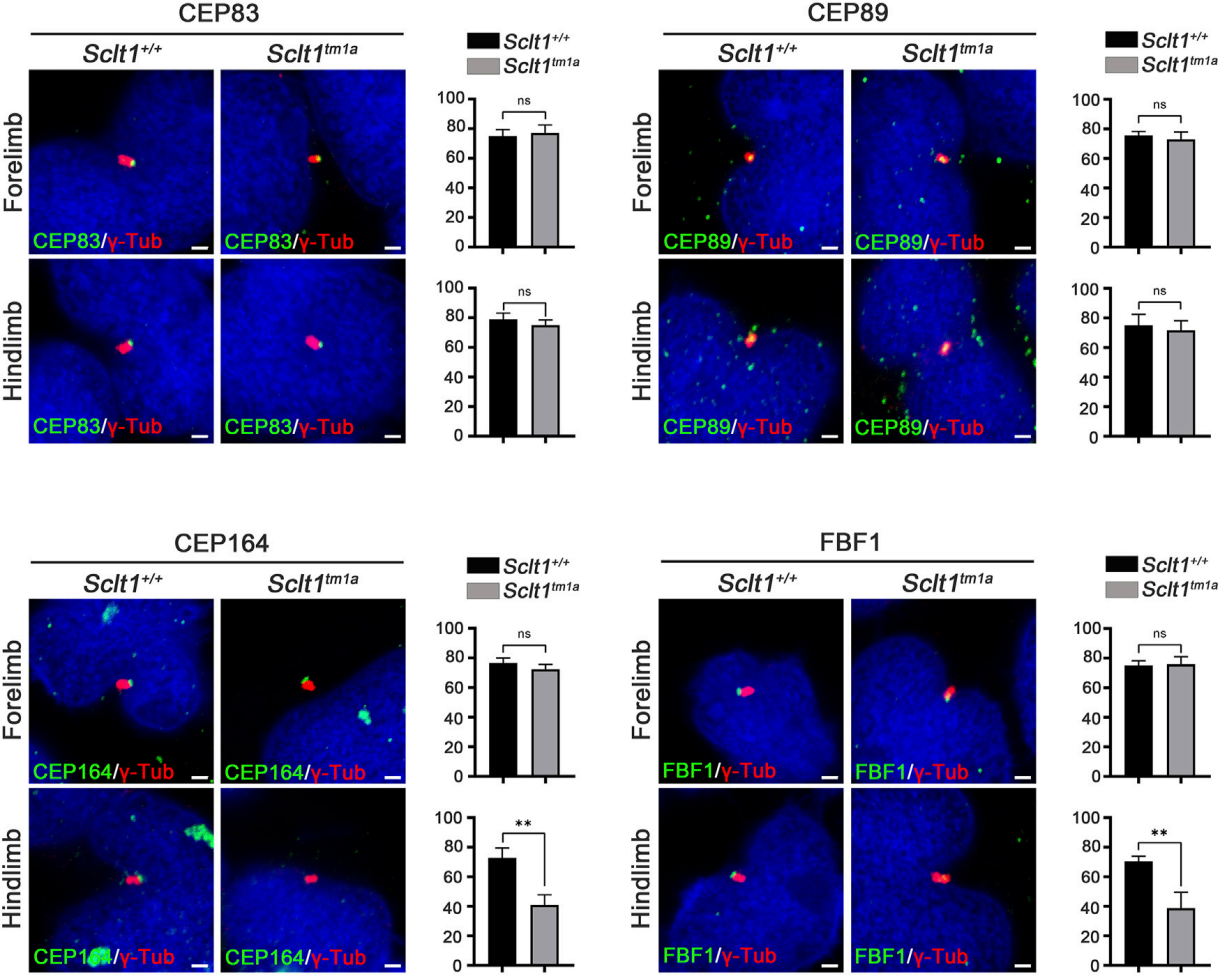
Sclt1 is involved in hierarchical DAP assembly during ciliogenesis of the hindlimb.To confirm SCLT1 mediated DAP assembly in mesenchymal cells obtained from E11.5 limb buds, we analyzed the localization of the DAP proteins such as CEP83, CEP89, CEP164, and FBF1 using immunofluorescent staining with indicated antibodies (green), and γ-Tub antibody (red) for basal body. Disruption of Sclt1 in mesenchymal cells prevents CEP164 and FBF1 recruitment to the basal body. Scale bars = 1 µm.

To initiate ciliogenesis, DAPs are assembled at the mother centriole in the centriole maturation stage. Sequential recruitment to the mother centriole is initiated by CEP83 localization to the centriole and subsequently recruited SCLT1 and CEP89 (Tanos et al., 2013). Also, SCLT1 further brings CEP164 and FBF1 to form the DAPs in cultured RPE1 cells. To verify this hierarchical DAP assembly in limb mesenchymal cells, we analyzed DAP proteins localization into the centriole in control and *Sclt1* mutant (Figure 2). Similar to the control hindlimbs, CEP83 and CEP89 were colocalized in the centriole in the mutant hindlimb. This result indicates that CEP83 and CEP89 are assembled in the centriole independent of SCLT1. However, loss of Sclt1 significantly disrupted centriolar localization of CEP164 and FBF1 in the mutant hindlimbs (Figure 2). We also analyzed DAP proteins expression levels in *Sclt1* mutant limb buds to rule out the possibility of reduced expression in hindlimb buds (Supplementary Figure S3). CEP83, CEP89, CEP164, and FBF1 protein levels were indistinguishable between the control and *Sctl1* mutant hindlimb buds. These results indicate that SCLT1 hierarchically recruits other DAP proteins such as CEP164 and FBF1 during DAP formation *in vivo*. They also suggest that incomplete formation of DAP by loss of Sclt1 would reduce ciliogenesis in a tissue-dependent manner.

### Sclt1 is required for ciliary vesicle docking to the centriole and subsequent tau-tubulin kinase-2 (TTBK2) recruitment and CP110 removal in the initiation of ciliogenesis

**FIGURE 3:**
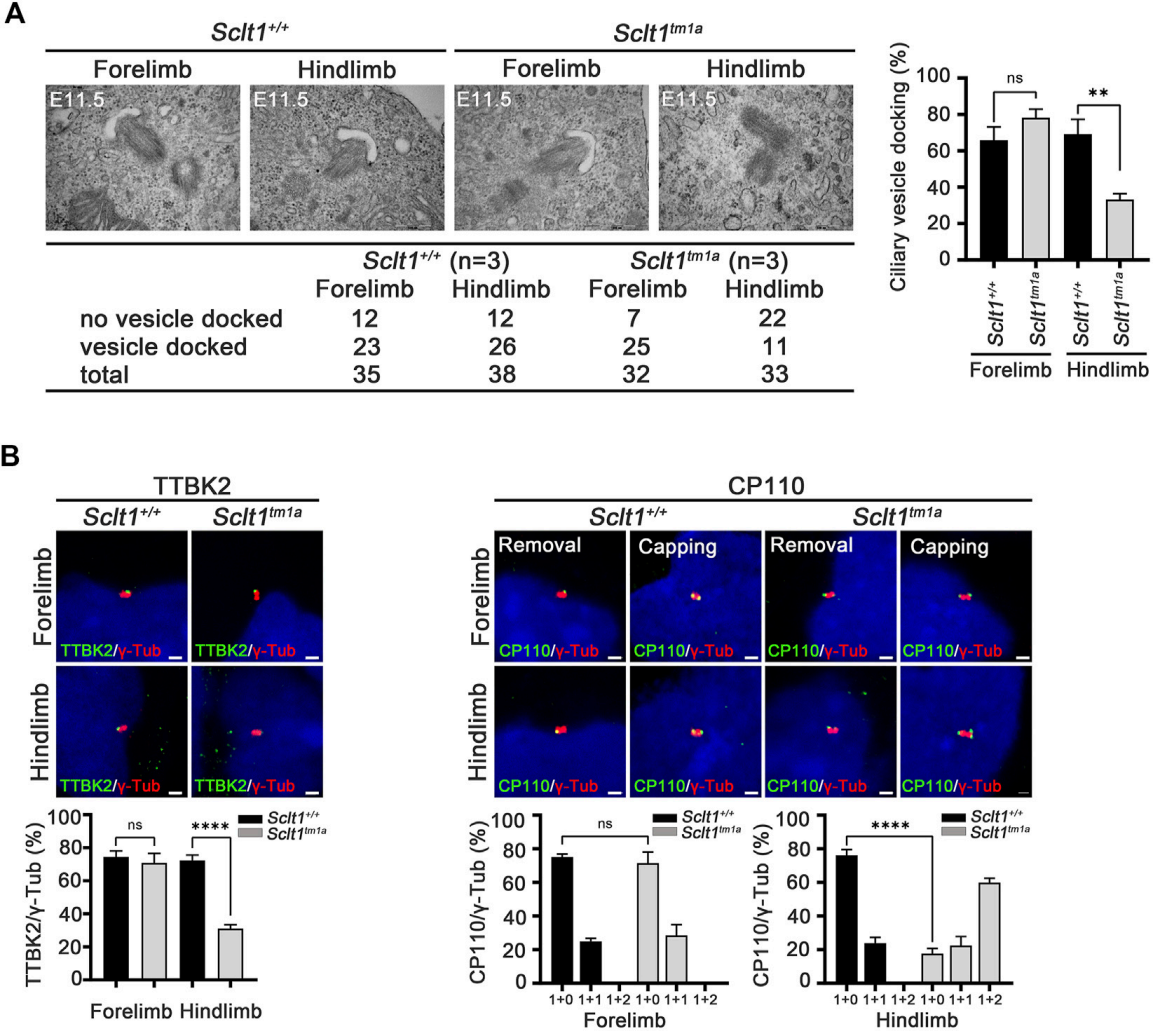
Sclt1 is required for initiation of ciliogenesis by anchoring ciliary vesicle through DAPs and subsequent tau-tubulin kinase-2 (TTBK2) recruitment **(A)** Transmission electron microscopy analysis of developing limb buds at E11.5 was carried out to identify the early stage of ciliogenesis, ciliary vesicle docking to the mother centriole. Loss of Sclt1 resulted in reduced ciliary vesicle formation in the distal end of the centriole. Quantitative analysis (right panel) was conducted to compare the extent of ciliary vesicle docking in limb buds from *Sclt1* mutant and wildtype control embryos. Error bars represent SEM (*n* = 3 independent samples; **p <0.01, Student′s *t* tests) **(B)** To examine the effects of Sclt1 on TTBK2 and CP110 localization in cultured limb mesenchymal cells, immunofluorescent images were taken from cells co-stained with anti-TTBK2 (green) or with CP110 antibodies (green), and with γ-Tub antibody (red). Representative images were shown in the top panel and quantitative results are presented in the bottom panel. Error bars represent SEM values and asterisks denote statistical significance according to Student′s *t* tests (****p <0.0001).

Ciliogenesis is a multi-step process that originates from the distal end of the centriole. During the early stage of cilia formation, distal appendage vesicles derived from Golgi and endosomes accumulate near DAPs to form preciliary vesicles (PCVs) that initiate ciliary extension (Ye et al., 2014; Wu et al., 2018). The absence of CEP83, a most proximal DAP protein, abolishes PCVs docking to the mother centriole in cultured cells (Joo et al., 2013). To better understand how Sclt1 disrupts ciliogenesis, we examined whether Sclt1 was involved in PCV docking at the initiation stage of ciliogenesis. Transmission electron microscopy analysis was performed for stage E11.5 limb buds (Figure 3A). We found that ciliary vesicle docking to centrioles in *Sclt1* mutant hindlimb was reduced by about 50% compared with wildtype hindlimbs or mutant forelimbs. These results suggest that Sclt1 also regulates PCV docking to the centriole during hindlimb development.

In the initiating stage, TTBK2 is a crucial factor for initiation of ciliogenesis by removing CP110 from the mother centriole. DAPs are involved in TTBK2 recruitment to the centriole to promote axoneme growth (Goetz et al., 2012; Lo et al., 2019). To examine whether the reduced ciliogenesis found in *Sclt1* mutant was caused by impairment of proper localization of TTBK2 and CP110 to the centriole, we used immunofluorescent co-staining of TTBK2 with γ-Tub antibody in the limb. We found that TTBK2 localization in the basal body was significantly diminished in the hindlimb of mutants, compared with the control (Figure 3B). CP110 has been known to play as a negative regulator for ciliogenesis and removal from the mother centriole is a prerequisite for the growth of primary cilia from the mother centriole. In the wildtype fore- and hindlimbs, most of the centrioles existed as single dots which indicate the removal of CP110 from the mother centriole (Figure 3B). In contrast, in the mutant hindlimbs, two or three dots were detected in centrioles indicating that both mother and daughter centriole retain CP110. These results support the notion that DAP is involved in TTBK2 recruitment into the mother centriole to remove the CP110 from the mother centriole at the initiation stage of ciliogenesis.

### DAPs in fore- and hindlimbs are formed differentially in a tissue-dependent manner

**FIGURE 4:**
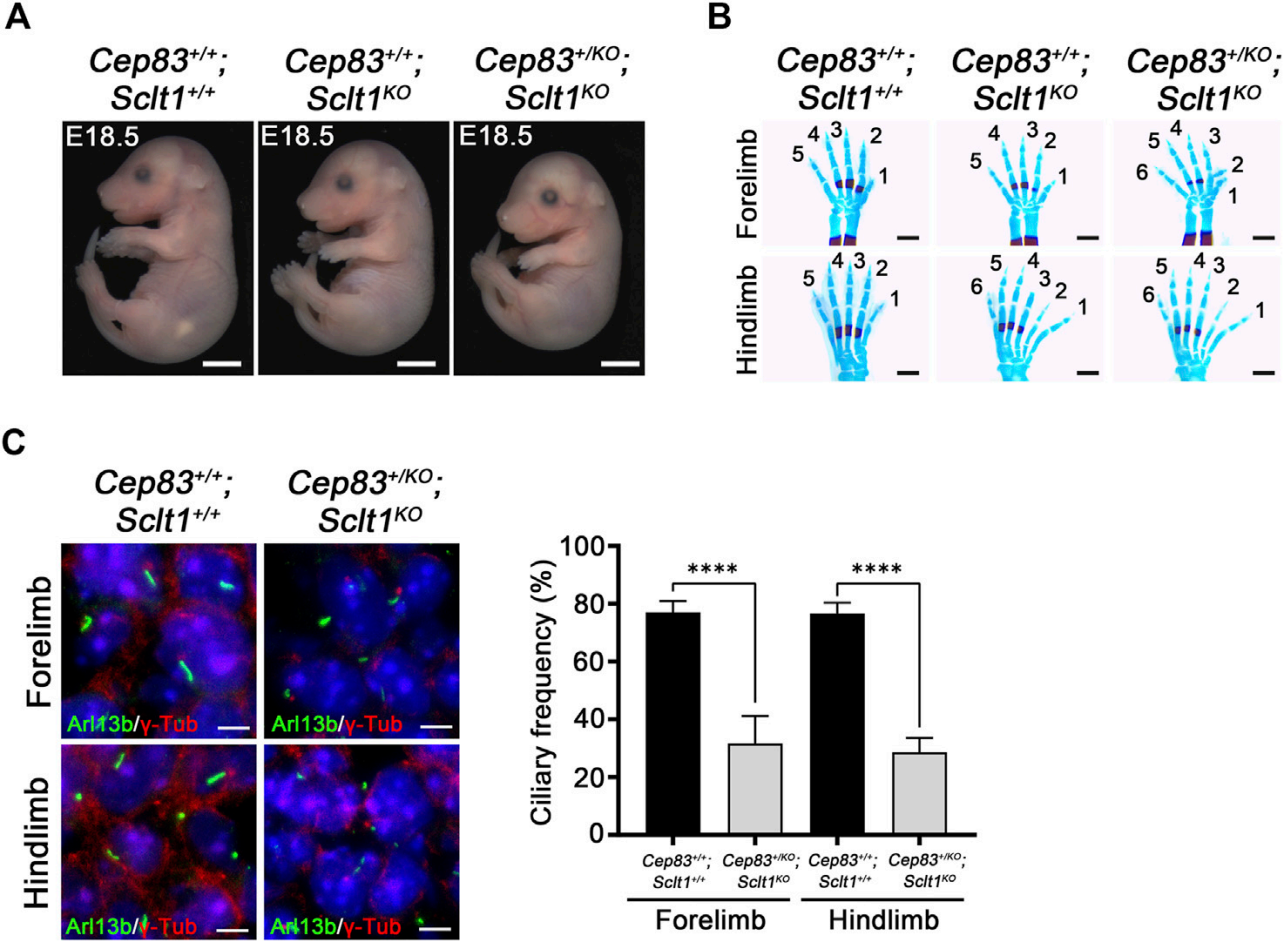
Heterozygosity of Cep83 in *Sclt1* mutant induces fore- and hindlimb polydactyly **(A)** Photomicroscopic image to compare the gross morphology of the *Sclt1* single mutant, *Sctl1* ^*KO*^, and heterozygous *Cep83* combined with *Sclt1* null mutant, *Cep83* ^*+/KO*^ *;Sctl1* ^*KO*^ with control embryos at E18.5. Scale bars = 2 mm **(B)** To evaluate the genetic interaction of Cep83 with Sclt1 in limb digit specification, we examined the digit patterning in the *Sclt1* single mutant, *Cep83* ^*+/+*^;*Sclt1* ^*KO*^; *Cep83* and *Sclt1* double mutant,*Cep83* ^*+/KO*^;*Sclt1* ^*KO*^; and control littermates, *Cep83* ^*+/+*^;*Sclt1* ^*+/+*^, by staining limbs with alcian blue/alizarin red. Heterozygous loss of *Cep83* gene with *Sclt1* homozygote mutant affects forelimb and hindlimb digit specification at E18.5. Scale bars = 500 µm **(C)** Immunofluorescent images were taken from cryosectioned E11.5 embryo limbs and analyzed with anti-Arl13b (green) and γ-Tub antibodies (red). Representative images were shown in the left panel and quantitative results presented in the right panel. Error bars represent SEM and asterisks denote statistical significance according to Student′s *t* tests (****p <0.0001).

To compare the role of each DAP component in mouse development, we also obtained the *Cep83* null mutant allele, *Cep83* ^*tm1(KOMP)/Tcp*^ (*Cep83* ^*KO*^) from IMPC. We found that homozygous *Cep83* mutant embryos display severely disrupted in early embryo development; a turning defect was present at stage E9.5 and premature death occurred around E9-10 (Supplementary Figure S4). Moreover, the digit patterning in the heterozygous embryo at E18.5 is indistinguishable from the wildtype control littermate (Supplementary Figure S4). We also analyzed ciliogenesis in the neural tube at E9.5 mutant embryos (Supplementary Figure S4). Disruption of Cep83 resulted in diminished ciliogenesis in developing neuroepithelial cells, compared with the control embryos. Due to premature death, we could not further analyze ciliogenesis in the mutant limb buds. To further examine the role of CEP83 in limb digit specification, we bred the *Sclt1* null allele, *Sclt1* ^*tm1d(EUCOMM)Hmgu*^ (*Sclt1* ^*KO*^) mice with *Cep83* heterozygotes to generate double mutants, *Cep83* ^*+/KO*^ *;Sclt1* ^*KO*^. The null allele of *Sclt1* was also indistinguishable from the tm1a allele with respect to hindlimb polydactyly (Supplementary Figure S5). In the absence of the *Sclt1* gene, we found no obvious difference in gross morphology in the *Cep83* heterozygous double mutant was observed as shown in Figure 4A. We performed the bone staining from E18.5 embryo limbs with alcian blue and alizarin red S to analyze the patterning of limb digits (Figure 4B). The *Sclt1* single mutant, *Cep83* ^*+/+*^ *;Sclt1* ^*KO*^, showed preaxial polydactyly in the hindlimb as shown in Figure 1D. Heterozygous loss of the *Cep83* gene in *Sclt1* homozygote mutant, *Cep83* ^*+/KO*^ *;Sctl1* ^*KO*^, affected forelimb as well as hindlimb digit specification (Figure 4B). We further examined ciliogenesis in *Cep83* and *Sclt1* double mutant limbs and compared them with control littermates. Lowering the gene dosage of Cep83 in the *Sclt1* mutant resulted in reduced ciliogenesis in both of limbs (Figure 4C).

**FIGURE 5:**
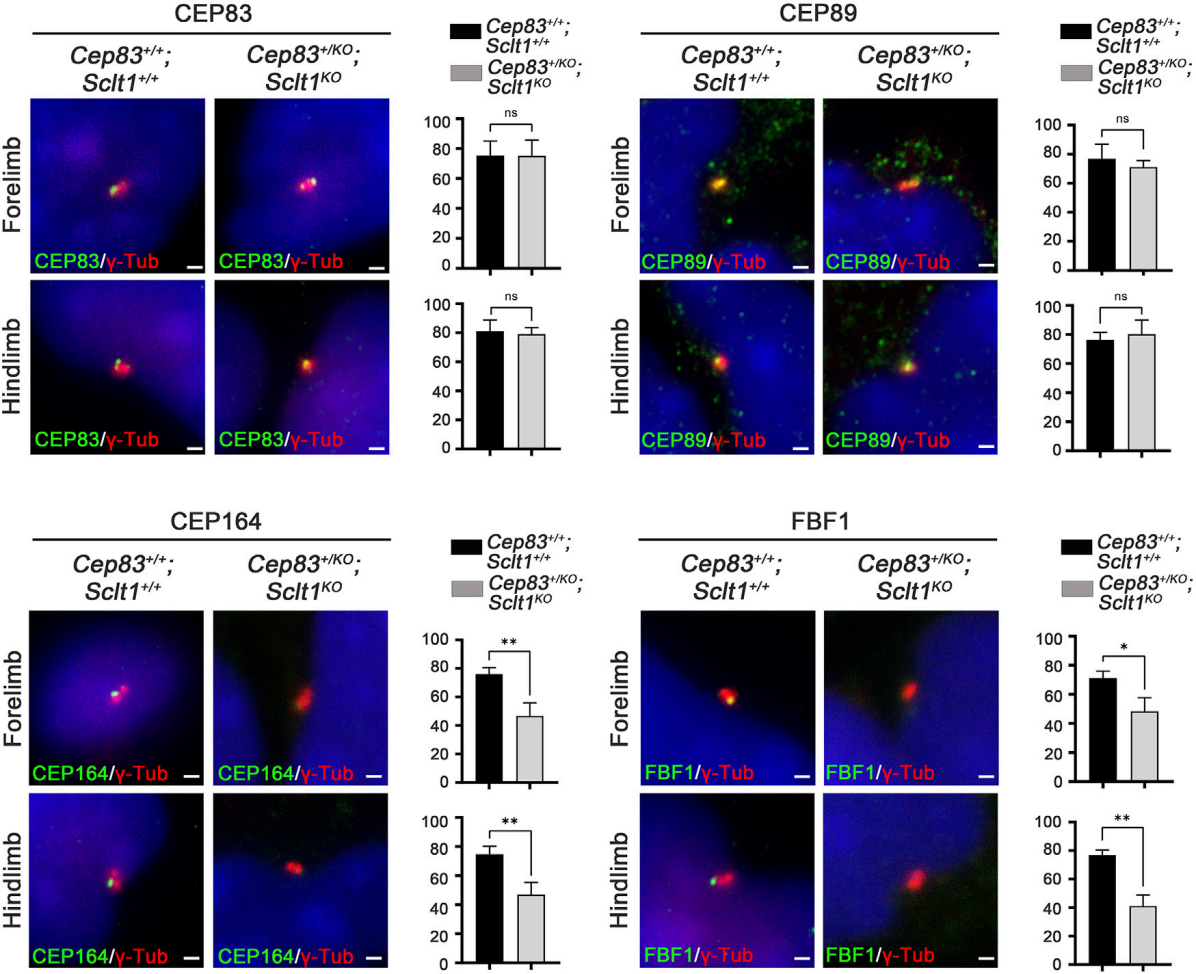
Heterozygosity of Cep83 in *Sclt1* mutant disrupts complete DAP formation in the forelimb Sclt1 mutant embryos had further disrupted forelimb digit patterning by heterozygous loss of Cep83. To confirm the role of Cep83 in complete DAP assembly in *Sclt1* mutant mice, limb mesenchymal cells were analyzed with DAP proteins specific antibodies to determine their proper localization in basal bodies. Scale bars = 1 µm. Quantitative analyses were done with more than 150 cells counted and statistical analysis with Student′s *t* tests were used (*p < 0.05; **p < 0.01).

Although Sclt1 exclusively affected DAP formation in hindlimb buds as shown in Figure 2, the heterozygous loss of *Cep83* in *Sclt1* mutants also disrupted ciliogenesis in the forelimbs. To further confirm the cause of reduced ciliogenesis in the forelimb by lowering Cep83 gene dosage, we examined DAP formation in the forelimb of the double mutant, *Cep83* ^*+/KO*^ *;Sclt1* ^*KO*^ (Figure 5). While CEP83 and CEP89 were colocalized with the centriole in wildtype control and double mutants, localization of CEP164 and FBF1 was impaired in double mutants compared with the wildtype. These results imply that Sclt1 is involved in DAP formation in fore- and hindlimb development. However, DAP composition in these appendages might be differentially formed during mouse limb development.

## Discussion

Regulation of ciliogenesis is especially important for vertebrate development and disruption of genes related to formation of the ciliary architecture causes multiple abnormalities in organ development (Badano et al., 2006; Reiter and Leroux, 2017). Sclt1 is the main component of the ciliary substructure, DAP (Tanos et al., 2013). In humans, mutation of SCLT1 causes ciliopathies and is subcategorized as an oral-facial-digital syndrome (Adly et al., 2014). Our mouse model for Sclt1 recapitulated the clinical features of human mutation of SCLT1. However, the developmental defects identified in the mouse model indicate that Sclt1 differentially affects organ development. The effect of Sclt1 in limb development was restrained in hindlimb development. Moreover, genetic interaction studies of another DAP component, Cep83 suggest that during limb patterning the integrity of DAPs in fore- and hindlimb mesenchymal cells might be distinct.

DAPs are transition fibers that anchor mature mother centrioles to the membrane. They are currently known to consist of seven genes including Cep83, Cep89, Cep164, FBF1, LRRC45, PIDD1, and SCLT1 (Tanos et al., 2013; Kurtulmus et al., 2018; Evans et al., 2021). DAP components were recently isolated from the biochemical study using cultured cells and were assembled in sequential recruitment to form a whole structure. Many studies of DAP participation in ciliogenesis have been performed in cultured cells but their roles *in vivo* are not determined. We provide the *in vivo* evidence in a mouse model that dysfunction of DAPs resulted in disruption of the development of multiple organs, such as the face, intestine, kidney, and limb. We also generated another DAPs component, *Cep83*, mutant mouse to compare with the *Sclt1* mutant. It also caused developmental disorders with embryonic lethality at the early prenatal stage. The *Cep83* mutant affected more early stages of development, such as body turning, at E9.5. These results suggest that Cep83 might have other functions different from Sclt1. It is also possible that the role of Sclt1 was restricted to specific tissues and other compensatory factors or programs contribute to DAP formation in different tissues. Mouse model-based findings revealed that other DAP genes also have distinctive phenotypic features. The *Cep164* KO-first mouse allele displayed holoprosencephaly, cardiac looping defects, and edema with lethality at E10.5 (Siller et al., 2017). Furthermore, multiciliated tissue-specific ablation of Cep164 causes defects in airway multiciliated cell differentiation. These findings indicate that results from mouse studies for core DAP genes are perplexing. Although some of the defects, such as cardiac looping, holoprosencephaly, polydactyly, and cystic kidney are common in major ciliary gene mutations, such as Kif3a or Ift88, the phenotypic spectrums of the mouse models for DAP genes are diverse. There might be tissue-specific different modes of DAP formation. However, it is also possible that there are diverse cilia-dependent and cilia-independent roles among DAP genes.

Assembly the DAP in the centriole is highly programmed and stepwise recruitment of DAP genes is required for proper DAP formation, followed by initiation of ciliogenesis in mammalian cultured cells (Reiter et al., 2012; Tanos et al., 2013; Yang et al., 2018; Bowler et al., 2019). Using a mouse model system, we also found a hierarchical recruitment program for DAP assembly. Loss of *Sclt1* only affected recruitment of other DAP proteins, such as CEP164 and FBF1 in affected tissues. In *Sclt1* mutants these events were only applied in limited cells which are palate, intestine, kidney, and hindlimb. Normally developing cells such as *Sclt1* mutant mesenchymal cells of the forelimb had normal generation of primary cilia. This result suggests that in the absence of Sclt1, the forelimb had alternative measures to form primary cilia. Interestingly, lowering the gene dosage for Cep83 in *Sclt1* mutant induced forelimb polydactyly. These findings suggest that Sclt1 in the forelimb involves in DAP assembly in an alternate manner. It is possible that the gene repertories for constructing DAPs are diverse in different tissues. Though Sclt1 is highly expressed in neuroepithelium cells, ciliogenesis and nervous system development were normal; The mutant mice of Cep83 and Cep164 showed reduced ciliogenesis and defects in neural tube development (Supplementary Figure S4 and (Siller et al., 2017)). These results also support the notion that diverse cell-type dependent programs for DAP assembly exist.

DAPs in cultured cells have been known to play a role in ciliary vesicle recruitment to the centriole and in TTBK2 recruitment to remove CP110 (Goetz et al., 2012; Schmidt et al., 2012; Oda et al., 2014; Čajánek and Nigg, 2014; Daly et al., 2016). The removal of CP110 initiates ciliogenesis by allowing axoneme growth from the mother centriole triplet microtubules. We found that Sclt1 was also involved in TTBK2 recruitment to the mother centriole, followed by CP110 removal *in vivo*. This *in vivo* study was the first to find that Sclt1 in DAP is important for TTBK2 recruitment during ciliogenesis. Intriguingly, we also observed that multiple CP110 positive dots are abnormally present in the basal body of about half of the *Sclt1* mutant cells. CP110 is previously known as a substrate for cell-cyle dependent CDK kinase and the abundance of CP110 is dependent on cell cycle (Chen et al., 2002). It also regulates centrosome separation in cultured cells. It might be possible that loss of Sclt1 in the hindlimb affects the proper cell-cycle dependent expression of CP110, resulting in dysregulation of the CP110 association with the basal body. It is interesting to know whether the reduced ciliogenesis in *Sclt1* mutant might be due to the improper centrosome metabolism.

A recent study from transgenic insertion allele for *Sclt1* found that loss of Sclt1 causes cleft palate, cystic kidney, and polydactyly (Li et al., 2017). The ciliogenesis of kidney in that allele is diminished, which leads to increase in kidney epithelial cell numbers by enhancement of TGFβ signaling. Our splicing mutant and deletion null alleles for *Sclt1* also consistently showed similar defects during embryo development. Human mutations of SCLT1 cause variable clinical features such as obesity, defects in craniofacial development, eye, and kidney, and they are not overlapping for each other (Adly et al., 2014; Katagiri et al., 2018; Morisada et al., 2020). Moreover, preaxial polydactyly had not been found in human mutations. Further studies for some form of heterozygous compound mutations in humans are required to determine whether the polymorphic variations in humans are causative for disease. Or it might need to compare how mutations are differentially affecting SCLT1 function. However, it also provides the some aspect of conserved role for Sclt1 as in human mutation which causes a ciliopathy and phenotypic features ranging from cleft palate to coloboma (Adly et al., 2014). This mouse model will be a valuable resource for delineating the pathogenesis of a rare human disease.

In summary, we generated *Sclt1* mutant mice that manifested similar developmental defects as in the human mutation. It also regulates complete DAP formation in the centriole to recruit TTBK2 for CP110 removal. Loss of *Sclt1* disrupted CP110 removal from the mother centriole and reduces ciliogenesis in the development of specific organs. During limb development, Sclt1 differentially affected digit patterning. While forelimb development was normal in the *Sclt1* mutants, hindlimb was selectively affected with preaxial polydactyly in *Sclt1* mutants. Interestingly, gene dosage effect of Cep83 on the *Sclt1* mutant was manifested as forelimb polydactyly. These findings imply that there are tissue-dependent and diverse DAP assembly programs rather than one common and unique program. Ciliopathies in humans and animal models suggest that diverse non-overlapping symptoms depending on affected ciliary genes. Understanding these pleiotropic clinical symptoms in ciliopathies are limited. Therefore, it is important to delineate the tissue-dependent DAP programs to understand the diverse ciliary gene function and related diseases.

## Data Availability

The original contributions presented in the study are included in the article/Supplementary Material, further inquiries can be directed to the corresponding authors.
