# Supplementary material for: Tissue-specific requirement of sodium channel and clathrin linker 1 (Sclt1) for ciliogenesis during limb development
Source: Front Cell Dev Biol. 2022 Nov 3;10:1058895. doi: 10.3389/fcell.2022.1058895 (PMC9669486; doi:10.3389/fcell.2022.1058895)
Supplement: Supplementary file 1 [file DataSheet1.PDF]

Supplementary Figures

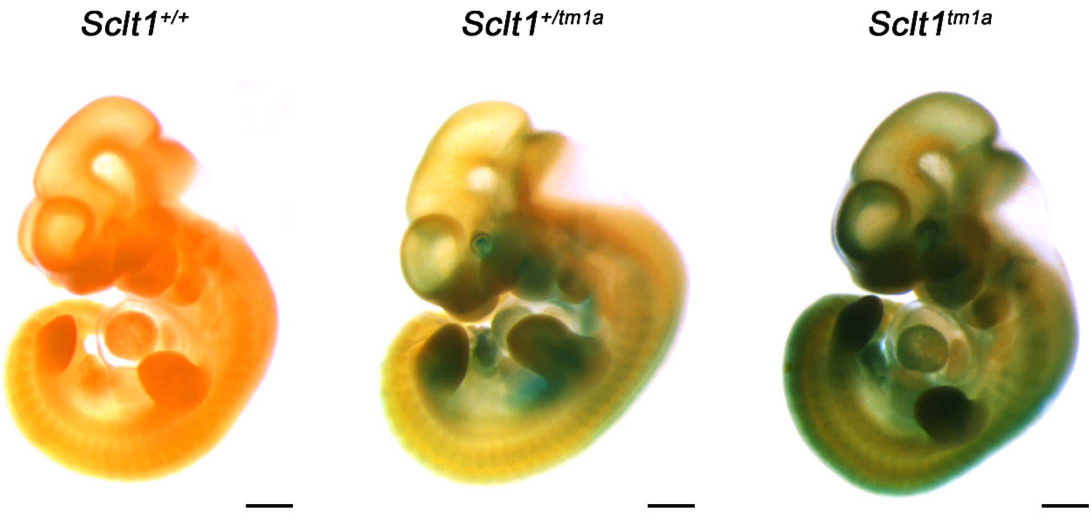

Supplementary Figure S1. Sclt1 is highly expressed in multiple tissues including limb buds.

Whole-mount X-gal staining of E10.5 heterozygous and homozygous alleles of Sclt1, *Sclt1*$^{+/tm1a}$, and *Sclt1*^{tm1a}, and control wildtype, *Sclt1*$^{+/+}$, was performed to visualize the tissue-specific distribution of Sclt1 gene expression. Sclt1 is highly expressed in the embryonic eye, neural tube, and limb buds at E10.5. Scale bars = 500 μm.

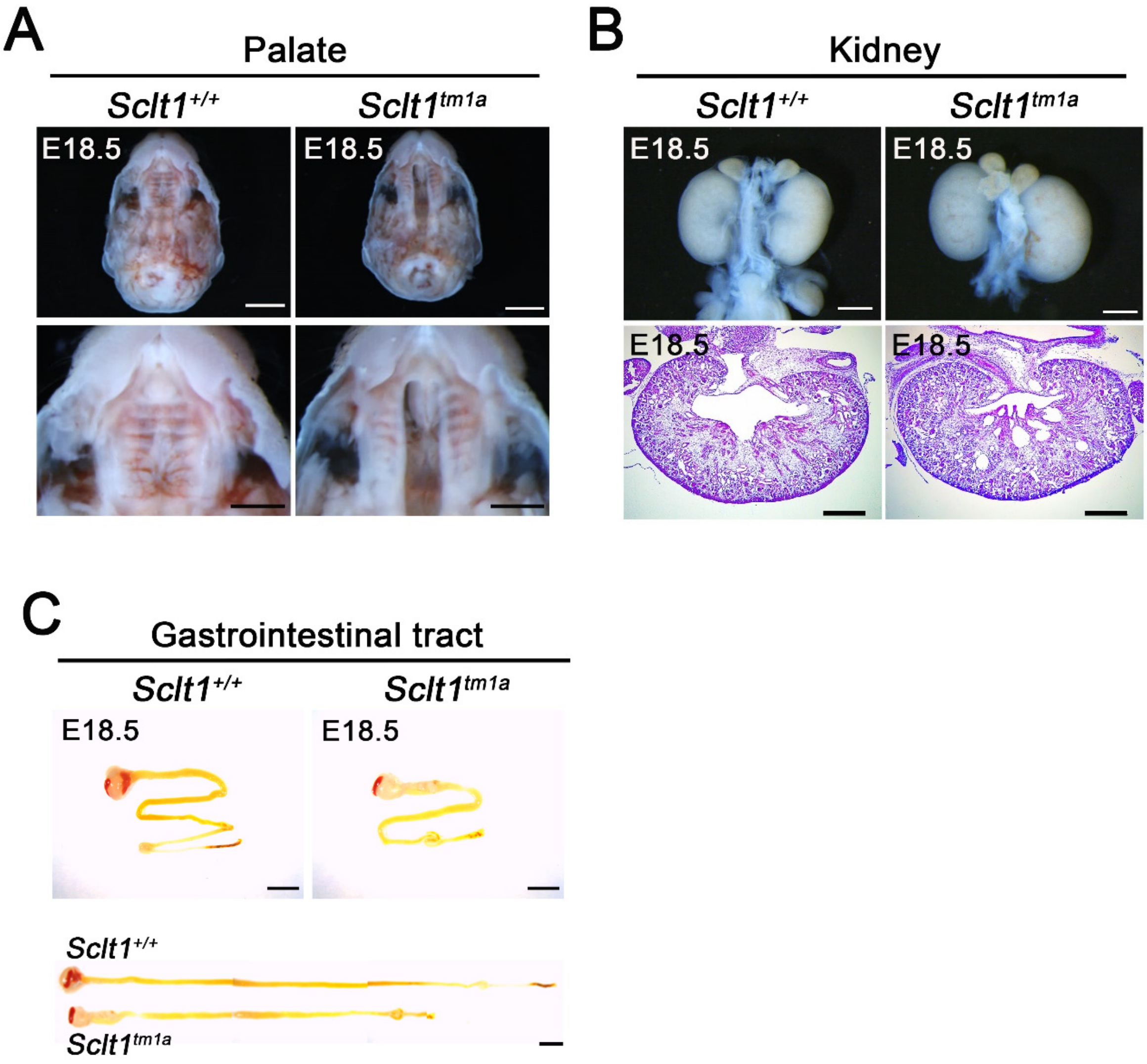

Supplementary Figure S2. Disruption of Sclt1 gene expression affects craniofacial, intestinal, and kidney development.

(A-C) Phenotypic analysis of *Sclt1*^{tm1a} mutant embryos was done to identify the organs affected by the dysfunction of Sclt1. Malformation of the palate, kidney and intestine were revealed as cleft palate, cystic kidney, and shortened intestine in E18.5 mutant embryos. Scale bars = 1 mm for top panels of A and C, and B; 2 mm for bottom panels of A and C.

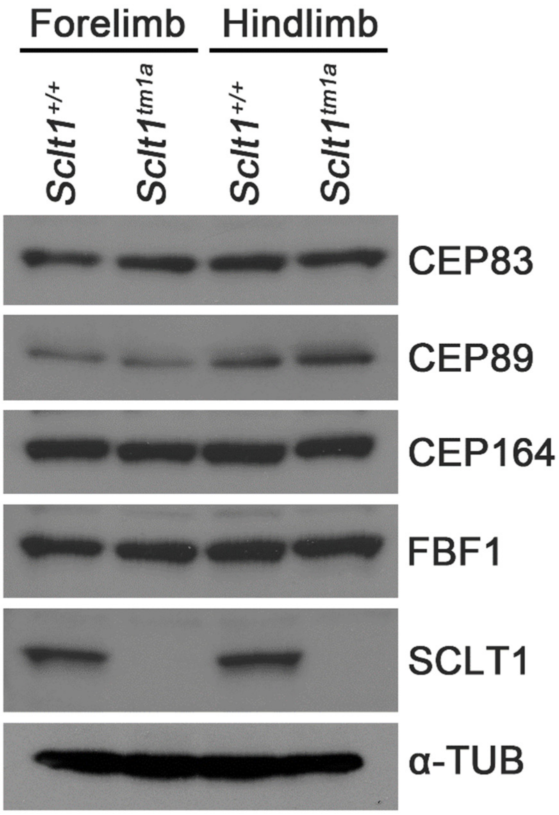

Supplementary Figure S3. DAP proteins were normally expressed in cultured primary mesenchymal cells of *Sclt1* mutant embryos compared to control littermates.

Expression levels of DAP proteins in *Sclt1*^{tm1a} limb buds-derived mesenchymal cells were examined by western blot analysis using gene-specific antibodies. Western blot analysis indicates that loss of SCLT1 expression in mutant does not noticeably affect the expression of other DAP proteins, such as CEP83, CEP89, CEP164, and FBF1. Alpha-tubulin (α-TUB) antibody was used for internal control.

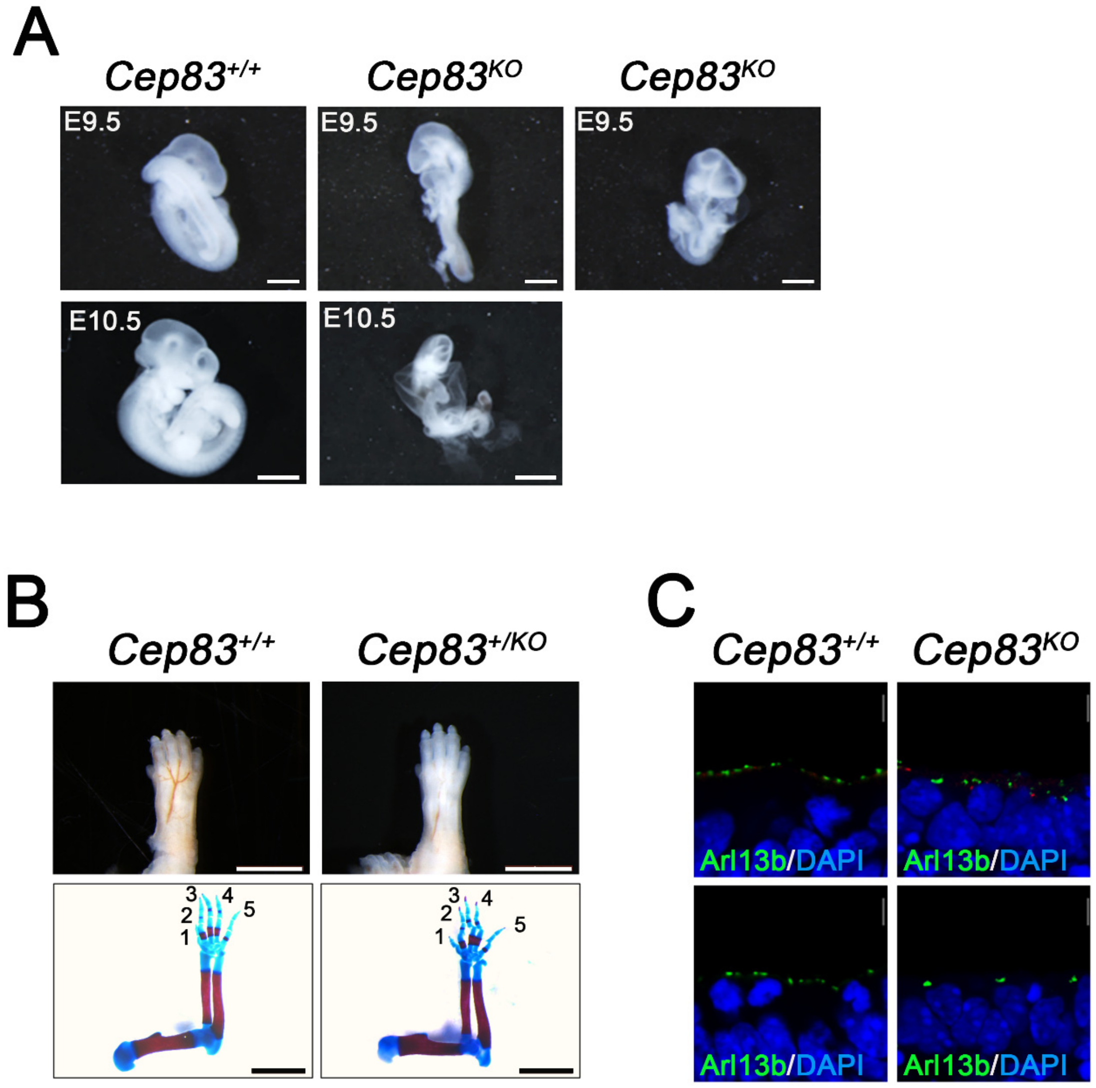

Supplementary Figure S4. Loss of Cep83 severely affects early embryonic development.

(A) Gross morphology of *Cep83* null mutant allele, *Cep83*$^{tm1(KOMP)/TCP}$ (*Cep83*^{KO}). The mutant embryo had a turning defect at stage E9.5. Scale bars = 0.5 mm. (B) Heterozygous *Cep83* allele, *Cep83*$^{+/KO}$ embryo developed with the normal specification of limb digits compared with control littermate at E14.5. Alcian blue and alizarin red staining was performed with E18.5 embryos. (C) Immunofluorescent staining of primary cilia in the neuroepithelial cells of E10.5 neural tube. Cryosectioned neural tube samples of *Cep83* mutant embryos were immunostained with anti-Arl13b antibody to detect primary cilia in neuroepithelial cells. Loss of Cep83 resulted in diminished ciliogenesis in the neural tube.

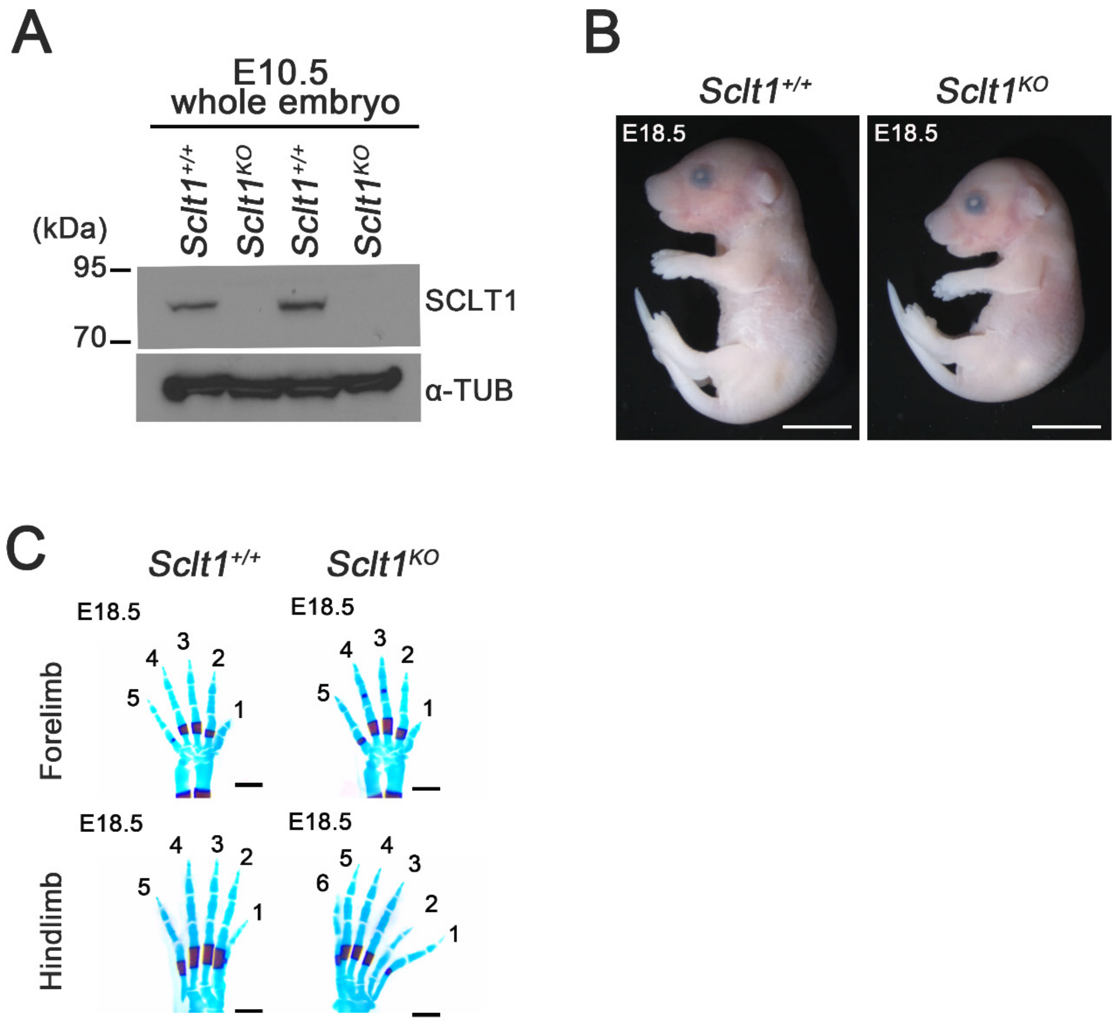

Supplementary Figure S5. Removal of the Sclt1 gene in mouse embryos displayed a preaxial polydactyly.

(A) The null allele of *Sclt1, Sclt1*^{KO}, was generated by deleting exon 5 of *Sclt1*^{tm1a} with E2a Cre-mediated recombination. Western blot analysis using SCLT1 specific antibody showed an undetectable signal in *Sclt1* mutant whole embryo extracts compared with control. (B-C) *Sclt1* mutant embryo was also indistinguishable from the tm1a allele with respect to hindlimb polydactyly. In the absence of the *Sclt1* gene, we found no obvious difference in gross morphology and limb digit patterning in E18.5 embryos. Scale bars = 2 mm and 0.5 mm, B and C, respectively.
